# Supplementary material for: Potential Role of Exosomes in the Chemoresistance to Gemcitabine and Nab-Paclitaxel in Pancreatic Cancer
Source: Diagnostics (Basel). 2022 Jan 23;12(2):286. doi: 10.3390/diagnostics12020286 (PMC8871170; doi:10.3390/diagnostics12020286)
Supplement: Supplementary file 1 [file diagnostics-12-00286-s001.zip › diagnostics-1548651-supplementary.pdf]

Supplementary Table S1. Candidate pancreatic cancer biomarkers and their potential application.

(Potential) Biomarker	Tissue	(Potential) Application	Reference
Ca 19.9<br>*FDA approved**	serum	Increased levels = Diagnosis<br>Reduced levels = Response to chemotherapy	[111,112]
CEA	serum	Increased levels =Diagnosis<br>Reduced levels = Response to chemotherapy	[111]
miR-181a-5p	plasma	Reduced levels = Predictive marker of longer PFS after FOLFIRINOX	[116]
miR-21<br>miR-155<br>miR-196a<br>miR-210	stool	High expression = Early Diagnosis	[113]
miR-21	PDAC tissue	High expression =Diagnosis<br>High expression = Predictive marker of shorter PFS/OS in gemcitabine-treated patients	[85]
hENT1	PDAC tissue	Low expression = Predictive marker of shorter PFS/OS in gemcitabine-treated patients	[29,114],
CES2	PDAC tissue	High expression = Predictive marker of longer OS FOLFIRINOX-treated patients	[115]
Osteonectin	Plasma	Increased levels = Diagnosis	[117]

* FDA approved for monitoring treatment effect in pancreatic cancer, high serum level is associated with advanced stage of the disease [118].
